# Evaluation of Osteogenic Differentiation of Bone Marrow-Derived Mesenchymal Stem Cell on Highly Porous Polycaprolactone Scaffold Reinforced With Layered Double Hydroxides Nanoclay

**DOI:** 10.3389/fbioe.2022.805969

**Published:** 2022-02-24

**Authors:** Seyedeh Elnaz Enderami, Seyedeh Sara Shafiei, Mehdi Shamsara, Seyed Ehsan Enderami, Abolfazl Rostamian Tabari

**Affiliations:** ^1^ Stem Cell and Regenerative Medicine Department, Institute of Medical Biotechnology, National Institute of Genetic Engineering and Biotechnology, Tehran, Iran; ^2^ National Research Center for Transgenic Mouse, National Institute of Genetic Engineering and Biotechnology, Tehran, Iran; ^3^ Department of Medical Biotechnology, School of Advanced Technologies in Medicine, Faculty of Medicine, Mazandaran University of Medical Sciences, Sari, Iran

**Keywords:** layered double hydroxide, polycaprolactone, nanoclay, bone tissue engineering, scaffold

## Abstract

In recent decades, bone tissue engineering has had an effective role in introducing orthopedic implants. In this regard, polymeric scaffolds reinforced with bioactive nanomaterials can offer great potential in tissue engineering implants for replacing bone loss in patients. In this study, the thermally induced phase separation method was used to fabricate three-dimensional highly porous scaffolds made of layered double hydroxide (LDH)/polycaprolactone (PCL) nanocomposites with varied LDH contents ranging from 0.1 wt.% to 10 wt.%. The Phase identification, morphology, and elemental composition were studied using X-ray diffraction, scanning electron microscopy, and energy-dispersive X-ray spectroscopy, respectively. Interconnected pores ranging from 5 to 150 µm were detected in all samples. The results revealed that the inclusion of LDH to PCL scaffold reinforced mechanical strength and compressive modulus increased from 0.6418 to 1.3251 for the pure PCL and PCL + LDH (1 Wt.%) scaffolds, respectively. Also, thermal stability, degradation rate, and biomineralization especially in comparison with the pure PCL were enhanced. Adhesion, viability, and proliferation of human bone marrow-derived mesenchymal stem cells (hBMSCs) seeded on PCL + LDH scaffolds were improved as compared to the pure PCL. Furthermore, the addition of LDH resulted in the increased mineral deposition as well as expression of ALP and RUNX2 osteogenic genes in terms of differentiation. All in all, our findings revealed that PCL + LDH (1 Wt.%) scaffold might be an ideal choice for 3D scaffold design in bone tissue engineering approaches.

## Introduction

Annually in the world, more than millions of people have been injured because of diseases, accidents, senility, and lesions, and unfortunately, a bone injury could not be repaired by itself (Black et al., 2015). There are different treatments for bone injuries, but one of the most applied methods for bone fractures and lesion repairing is bone tissue engineering (Amini et al., 2012). In the last decades, tissue engineering has had an influential role in introducing bone bioimplants. The essential parts of tissue engineering are scaffold structure, stem cells, and signaling factors. Scaffolds have a significant role in tissue engineering; they provide an appropriate environment for cells to proliferate, differentiate, migrate, adhere, grow, and extend. Moreover, signaling growth factors induce the differentiation into the targeted cell lines (Shafiei et al., 2016; Hosseini et al., 2019). There are several techniques for scaffold fabrication, such as electrospinning, solvent casting, extrusion, freeze-drying, gas foaming, milling, 3D printing, and thermally induced phase separation (TIPS) (Shafiei et al., 2016; Gandolfi et al., 2019). TIPS method provides a scaffold with high porosity (>90%) and interconnected pore structure under low temperature. By changing the temperature in this method, a multiphase system is formed by the de-mixing of a homogeneous polymer solution. Formation of porous structure with a variety of pore dimensions is the feature property of this technique (Conoscenti et al., 2017; Gandolfi et al., 2019). In bone tissue engineering, scaffolds should be biocompatible, biodegradable, and bioactive with adequate mechanical and thermal properties (Guarino and Ambrosio, 2010). In recent decades, polycaprolactone (PCL) is one of the profitable and applied polymers with FDA approval and has been used in scaffold fabrication for tissue engineering applications. PCL is biodegradable polyester, and its melting point is about 60°C, but PCL is hydrophobic in nature that causes low cell adhesion. Other disadvantages include slow degradation rate, low thermal stability, and weak mechanical characteristics (Li et al., 2014; Mohammadi et al., 2017). Layered double hydroxide (LDHs) are well known as anionic nanoclays, and have been investigated in the fields of healthcare, drug delivery systems, molecular therapy, catalyst synthesis, and biomedicine. The existence of magnesium and calcium ions within the LDH chemical structure makes it a suitable candidate for bone tissue engineering applications. The idea of using LDH in combination with other polymers has shown their synergistic effects for various biomedical applications (Yang et al., 2010; Fayyazbakhsh et al., 2012). Recently, there are a series of reports on the research of polymer/LDH nanocomposites. It is worth mentioning that most of the studies on PCL/LDH focus on reinforcing the mechanical and biological properties. In addition to enhancing bioactivity, cell attachment, viability, and differentiation, the addition of LDH to the highly porous PCL could compensate for the decrease in the mechanical stability because of the presence of high porosity (>90%) produced by the TIPS method. Therefore, synergistic strategies and techniques can overcome challenges that arise in the field of biocomposites. However, to the best of our knowledge, the study of highly porous PCL/LDH nanocomposites fabricated by the TIPS method has not been studied so far.

In the current study, we have used the TIPS method for reinforcing PCL by different LDH contents ranging from 0.1 wt.% to 10 wt.%. The physical, mechanical, and biological features of nanocomposite scaffold containing LDH nanoclay were investigated. The purpose of this research is to design a highly porous PCL + LDH nanocomposite scaffold with optimum physiochemical and biological features, suitable for bone tissue engineering applications. Furthermore, a highly porous scaffold with the optimum property was selected to assess the osteogenic differentiation potential.

## Material and Methods

### Synthesis of Mg/Al LDH Nanoclay

One of the most conventional methods for synthesizing LDH is co-precipitation. Briefly, aluminum chloride hexahydrate (AlCl_3_·6H_2_O, 99.9%) and magnesium chloride hexahydrate (MgCl_2_·6H_2_O, 99.9%) solutions have been mixed with a 2:1 M ratio as the basic components. The sodium hydroxide solution (0.2 M) was added to the mixture while the pH was maintained at 10 during the reaction. Ultimately, the precipitate was centrifuged for 15 min at 3,900 rev/min and rinsed several times with deionized water until the pH reached 7, then dried at 90°C overnight (Bravo-Suárez et al., 2004; Shafiei et al., 2016).

### Scaffold Fabrication by TIPS Method

The porous nanocomposite scaffolds were prepared by the TIPS method. First Poly ( ε -caprolactone with Molecular weight of 70,000–90,000 g/mol was dissolved in 1,4-dioxan (DIOX, 3.5% wt/vol) under stirring at room temperature. LDH powder was sonicated for 1 h in DIOX to obtain homogenous LDH dispersions with different concentrations of 0.1,1, and 10 wt.%. Also, dispersed LDH was added to PCL solution by different LDH nanoclay ranging from 0.1 wt.% to 10 wt.%. The homogeneous mixture was dispersed by sonicating for 1 h using the ultrasonic processor UP50H (Hielscher, 50 W, 30 kHz). After mixing, contents were placed into glass test tubes of 10 mm in diameter and cooled at −18°C. After 18 h, frozen samples were kept at −70°C for 72 h and completely dried in a freeze dryer for 24 h (Freeze-dryer, Cool Safe TM; Scanvac, Lynge, Denmark). Four samples (diameter 10 ±1 mm, thickness 100 ± 10 mm) per composition were prepared (PCL, PCL + LDH (0.1 Wt. %), PCL + LDH (1 Wt.%), PCL + LDH (10 Wt.%)).

### Structural and Morphological Characterization

The phase identification and structural characterization of as-synthesized LDH were validated using XRD through a D4 Bruker (CuKα radiation (λ = 1.5406 Å)) through the 2Ѳ range of 5–60° at a scan rate of 0.02°min^−1^ in Guinier geometry. The utilized current and voltage were 30 mA and 40 Kv, respectively. The morphology and microstructure of porous nanocomposite were assessed using scanning electron microscopy (SEM) at 20 kV (VEGA, TESCAN, Czech Republic). The porous scaffolds were dried entirely and coated with a paper-thin layer of gold (EMITECH K450X, UK). Samples were horizontally cut to exhibit their cross-section. The average pore size of samples was estimated by employing the ImageJ program. The distribution of LDH contents, aluminum, and magnesium within nanocomposite scaffolds was detected by an energy-dispersive X-ray (EDX) test (ZEISS, Germany). EDAX was carried out at accelerating voltages 1–20 kV and 280 × magnification.

### Mechanical Characterization

Mechanical characterization of porous scaffolds was determined using the universal testing machine (SMT_20, SANTAM, Tehran, Iran). Scaffolds were sectioned to cylindrical specimens (10 ± 1 mm in diameter and 10 mm in thickness) and then were compressed under a load cell of 200 N pressures and a loading rate of 1 mm/min until fracture. The test was performed at room temperature. At least, three individual measurements were recorded for each sample (*n* = 3).

### Porosity Measurement

The liquid displacement technique was employed to evaluate the porosity of scaffolds. Ethanol as the displacement liquid was selected due to easy and quick infiltration through the pores without changing the scaffold structure. In brief, entirely dried samples were dipped in absolute ethanol for 36 h followed by removing excess liquid on the surfaces. Specimens were then weighed, and scaffolds porosity was calculated by the equation:

$$\text{Porosity}\left(\%\right)=\left[\frac{\left({\text{W}}_{2}-{\text{W}}_{1}\right)}{\rho \text{V}}\right]\times 100\%$$

where W_1_ and W_2_ refer to the weights of scaffolds before and after immersion in ethanol, respectively, ρ shows the density of absolute ethanol, and V represents the volume of the samples (Shafiei et al., 2016).

### Thermal Characterization

The TGA of all samples was conducted on a thermogravimetric analyzer (Q600, TA, United States) and DSC (Q600, TA, United States) under Ar and Zero Air. The rate of 20 °C/min was applied until up to 800°C.

### *In vitro* Degradation

Porous samples were cut into cylindrical shapes (10 ± 1 mm in diameter and 10 mm in thickness) and incubated in 0.5 M sodium hydroxide (NaOH) solution at 37°C for accelerated degradation conditions (*n* = 3). The NaOH solution was gently pipetted out at varying time intervals, and the porous scaffold was rinsed several times with purified water. After that, the scaffolds were frozen using liquid nitrogen.

### *In vitro* Biomineralization

A simulated body fluid (SBF) solution was used as an effective way to assess the ability of scaffolds to form a mineralized matrix. Porous scaffolds containing varying amounts of LDH (0, 0.1, 1, and 10 Wt. %) were cut into 10 ±1 mm in diameter and 10 mm in thickness specimens. At 37°C, the samples were incubated with 3 ml of SBF solution. The SBF solution was renewed every 2 days while the samples were incubated at 37°C for 7 days. After that, the samples were gently rinsed with distilled water and then freeze-dried. The morphological and chemical compositions of the mineralized samples were studied using SEM and EDX techniques.

### Cell Culture Study

Mesenchymal stem cells (human bone marrow-derived mesenchymal stem cells (hBMSCs)) were supplied from the Bonyakhte cell bank of Iran. Cells were cultured and held at 37°C in a humidified environment with 5% CO_2_ in T-25 cm^2^ flasks containing high glucose DMEM supplemented with 10% FBS [heat-inactivated,Gibco] and 1% pen/strep [100 U/100 mg/ml; Gibco]. Every two or 3 days, the medium was modified. As the cells reached 80% confluency, they were detached with 0.5 g/ml trypsin and counted with a hemocytometer (density of ∼10^6^ per flask). Cells were used for further tests at the fourth passage At least, three individual measurements were recorded for each sample (*n* = 3).

### Alizarin Red Assay

The hBMSCs at passage four were used for further analysis. Alizarin red staining, differentiation, and other assessments were investigated after 14 days of cell seeding. Dulbecco’s modified Eagle medium (DMEM) combined with 10% FBS, 10 nM beta glycerol phosphate) Sigma_Aldrich), 0.05 mM ascorbic acid (Darou Pakhsh), and 0.1 M dexamethasone (Iran Hormone) were used as osteogenic differentiation media. Through Alizarin Red Staining, the mineralized matrix created by hBMSCs was determined qualitatively and quantitatively. Two weeks after seeding cells on scaffolds, the differentiation culture medium was removed, and the scaffolds were washed twice with PBS. A solution of Alizarin Red S (2% (w/v), ARS; Sigma Aldrich) with a pH of ∼4.2 was applied to the scaffolds, covering the whole surface. The surplus ARS was washed with distilled water. The inverted optical microscope was used to take photographs of the ARS results. For quantification, 800 µl of 10% (v/v) acetic acid was transferred to each well. The plate was incubated at room temperature for 30 min and was shaken before moving to a 1.5 ml microcentrifuge tube with a wide-mouth pipette. The mixture was then covered with 500 µl of mineral oil (Sigma–Aldrich) and heated to precisely 85°C for 10 min. After vortex mixing for 30 s, they were put into the ice for 5 min. Following centrifuging the slurry at 20,000 RPM for 15 min, 500 µl of the supernatant was transferred to a new 1.5 ml microcentrifuge tube. According to neutralizing the acid, 200 µl of 10% (v/v) ammonium hydroxide was added. In a 96-well shape, aliquots (150 µl) of the supernatant were read by STAT FAX 2100, USA Microplate Reader in triplicate at 405 nm.

### Osteogenic Differentiation Study

First, Scaffolds were sterilized by dipping them in 70% ethanol for 20 min at 25°C. After UV irradiation, samples were washed three times with PBS. The hBMSCs (5 × 10^4^) were seeded on the scaffolds, and two groups of experiments were organized: 1) the pure PCL as control, 2) PCL + LDH (1 Wt. %) (as an optimum scaffold). The osteogenic medium was used for both groups.

### Cell Morphology Studies

Sterilized scaffolds with a diameter of 4 mm and a thickness of 1 mm were placed into wells of a 96-well plate. In a 200 µl standard growth medium, cells were seeded at a density of 10,000 cells per well on the scaffolds and incubated for 24 h. At each time point, the scaffolds were carefully washed three times with PBS and then fixed for 5 h in a 2.5% glutaraldehyde solution. The scaffolds were then dehydrated in a serial dilution of solutions before being air-dried at 25°C. The morphological features of cells were studied using SEM analysis.

### MTT Assay

hBMSCs were cultured with the seeding density of 15 × 10^3^ on the scaffolds, and the pure PCL was used as a control in the MTT (3-(4,5-dimethylthiazol-2-yl)-2,5-diphenyltetrazolium bromide (Sigma-Aldrich)) assay. After seeding, MTT solution (5 mg/ml) was applied to each well on days 1, 3, and 5 and incubated for 4 h at 37°C and 5% CO_2_. The supernatants were then removed, and the formazan crystals were solved in dimethyl sulfoxide (Sigma-Aldrich), and their optical densities were recorded at 570 nm using STAT FAX 2100, USA Microplate Reader.

### Real-Time PCR

The two bone-related genes, Runx2 and ALP, were quantified using real-time RT-PCR at days 3, 7, and 14 after cells seeding on the pure PCL as a control sample and PCL + 1% LDH as an optimal scaffold and were presented as the relative expression fold change. The manufacturer’s protocol was used for total RNA Extraction. For the synthesis of first-strand cDNA, the PCR conditions were as follows: 95°C for denaturation (30 s), 59°C for annealing (30 s), and 72°C for elongation (30 s) 35 cycles in which Random hexamers (Add bio, Korean) and reverse transcriptase (Add bio, Korean) were utilized. SYBR Premix-Ex-Taq master-mix) Yekta Tajhiz Azma) was used for real-time RT-PCR by the Mic qPCR machine, and the gene expression was calculated in comparison with GAPDH as an endogenous control.

### Statistical Analysis

The Faffle formula was used to indicate the data derived from real-time RT-PCR. Data were presented as the mean ± standard deviation (SD) and one-way analysis of variance (ANOVA) was used for comparison using graph pad prism 5 software (version 5.01). The student’s t-test was applied to make the quantitative analysis, and a *p*-value was used to show significance.

## Results

### LDH Characterization

**FIGURE 1:**
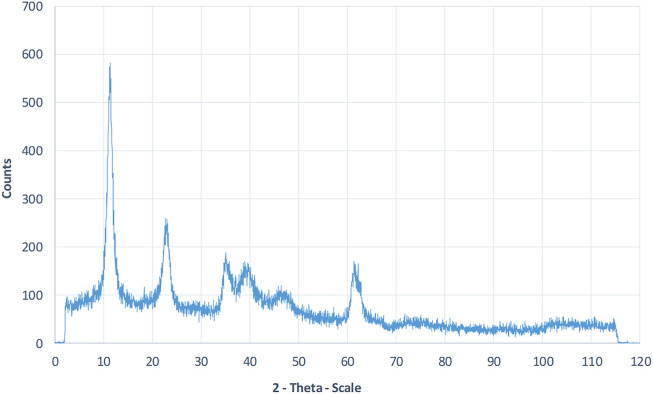
XRD patterns Analysis of synthesized LDH powder. The significant peak of LDH occurs at 2Ѳ = 11.2°, which conforms to the JCPDS 00-035-0965 standard pattern.

LDHs were synthesized using the co-precipitation process described previously. LDH was successfully synthesized with regard to the XRD data result. The acquired X-ray diffraction spectrum of LDH powder is shown in the Figure 1. The significant peaks of LDH conform to the JCPDS 00-035-0965 standard pattern (Kino et al., 2017).

### Morphological Characterization of Nanocomposite Scaffolds

**FIGURE 2:**
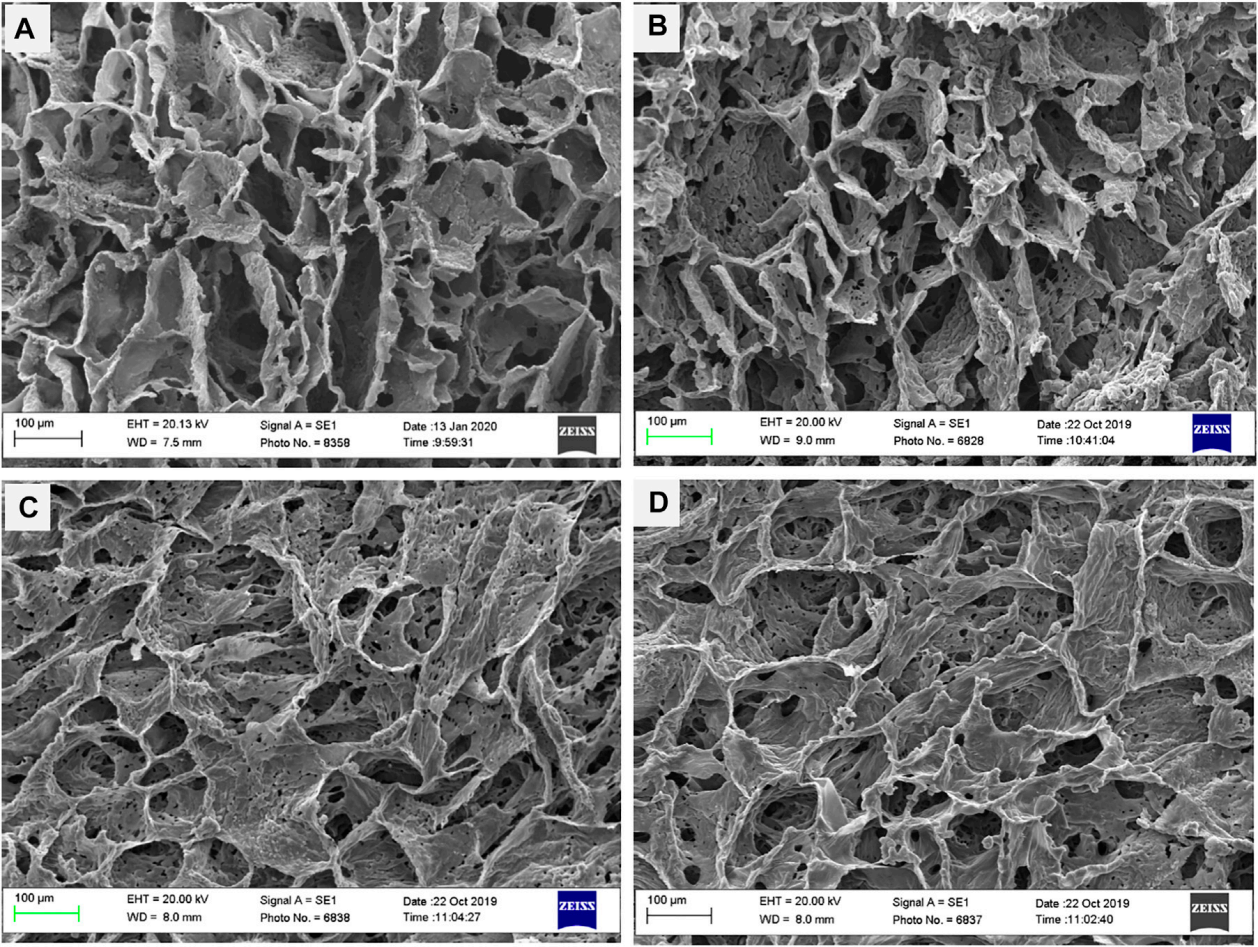
SEM morphology of nanocomposite porous scaffolds prepared by TIPS method; **(A)** PCL, **(B)** PCL/0.1 wt% LDH, **(C)** PCL/1 wt% LDH, **(D)** PCL/10 wt%LDH (scale bars represent 100 μm).

**FIGURE 3:**
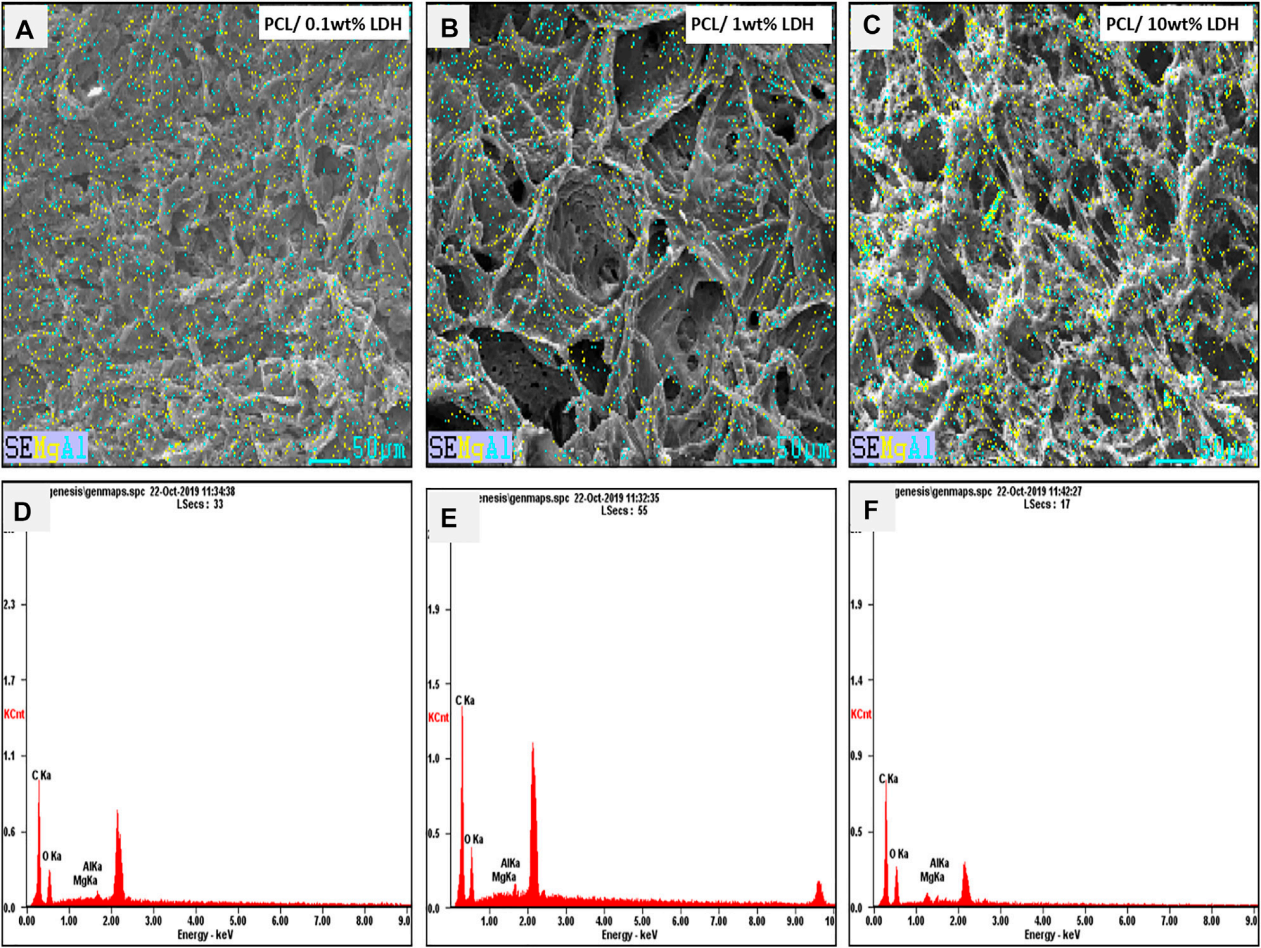
The Element map of aluminum (blue dots) and magnesium (yellow dots) within the PCL/LDH scaffold, demonstrating EDX analysis of samples. The spectrum displays peaks of carbon, oxygen, magnesium, and aluminum in each nanocomposite scaffold. **(A,D)** PCL/0.1 wt% LDH, **(B,E)** PCL/1 wt% LDH, and **(C,F)** PCL/10 wt% LDH.

As reported in other studies, choosing the series of porosities from macro to micro and nano in size is critical for designing scaffolds that foster excellent cell migration and penetration while facilitating the nutrient transfer. Figure 2, displays SEM micrographs of nanocomposite scaffolds with pore sizes ranging from 5 to 150 µm. Also, many smaller or larger open micropores were detected as well. Pore sizes in the cortical bone range from 10 to 50 µ and 100–300 µ which are crucial for vascularization, cell colonization, and tissue formation. EDX analysis was conducted to evaluate the elemental composition of nanocomposite scaffolds and to determine the distribution of nanoparticles within the PCL. The significant peaks for carbon and oxygen are attributed to the basic elements of PCL (Figure 3). In addition, the EDX spectra of PCL + LDH 0.1, 1, 10 wt. % showed two weak peaks relating to magnesium and aluminum elements. The existence of a strong peak in the spectrum is linked to gold. Furthermore, the EDX elemental map confirmed that magnesium and aluminum were distributed uniformly throughout the nanocomposite scaffolds.

### Porosity Measurement

The porosity percentages of PCL nanocomposites and the pure PCL scaffolds are shown in Table 1. The results revealed that porosity values ranged from 90 to 95 percent and the percentage of porosity did not remarkably change while the weight percentage of LDH increased. As a result, adding LDH to PCL polymers had no discernible influence on their porosity. The porosity percentage did not have a significant influence on their connectivity, as shown by the SEM analyses in Figure 2. It should be mentioned that these highly porous scaffolds are produced using the TIPS method.

**TABLE 1:** Values of mechanical properties and porosity measurments of scaffolds.

Scaffold	Porosity (%)	Compressive modulus (MPa)	Max displacement (mm
PCL	94.5	0.6418	10.0681
PCL/0.1% LDH	95	0.7637	8.9028
PCL/1% LDH	96	1.3251	7.8588
PCL/10% LDH	94	0.8783	9.3385

### Mechanical Properties

**FIGURE 4:**
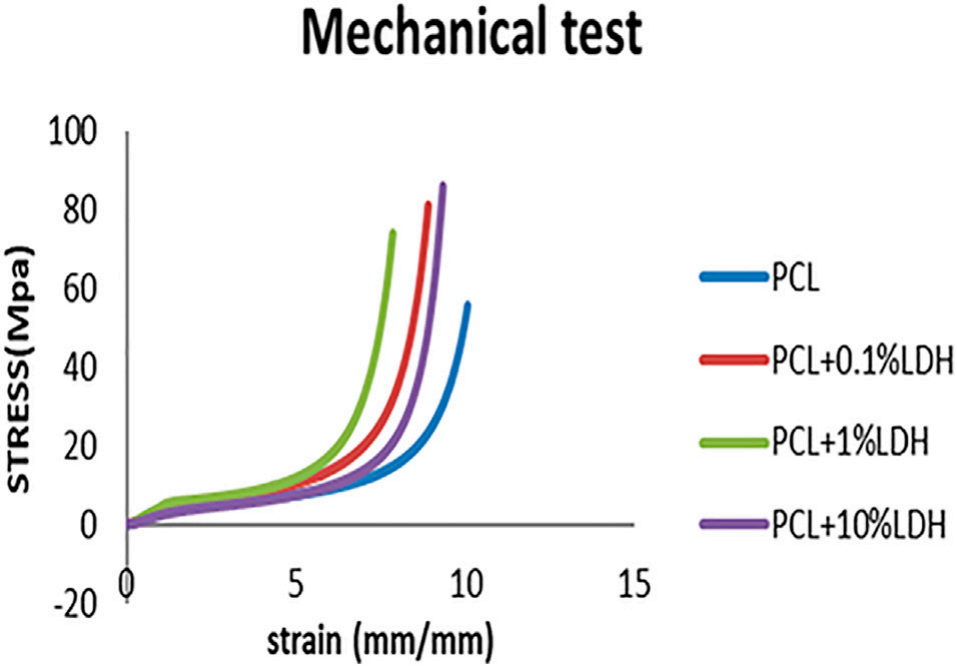
PCL scaffold stress-strain curves compared to PCL/0.1 wt% LDH, PCL/1 wt% LDH, and PCL/10 wt% LDH scaffolds.

Mechanically bolstered porous scaffolds are essential in bone implants because they provide a temporary framework for cell proliferation while enduring *in-vivo* stresses and pressures during bone remodeling. The stress-strain curves for samples are presented in the Figure 4. The compressive strength of the pure PCL was 0.562 MPa, while young’s modulus was 0.641 MPa. When a slight amount of LDH (0.1 Wt. %) was added, the compressive strength increased to 0.693 MPa, and Young’s modulus increased to 0.793 MPa. As the LDH concentration increased to 1 Wt. %, a significant elevation in compressive force to 1.041 MPa and Young’s modulus to 1.325 MPa was observed. On the other hand, when the LDH content was increased to 10 Wt.%, Young’s modulus, and compressive strength were significantly diminished to 0.804 and 0.878 MPa, respectively. Based on the results, it is reasonable to conclude that among the scaffolds, PCL + LDH (1 Wt.%) has the highest young’s modulus and compressive strength (Standard deviation: 0.2 and *p* ≤ 0.0001). Young’s modulus of the PCL scaffolds increases as the percentage of LDH nanoparticles rises. Although the compressive strength and Young’s modulus were reduced in PCL + LDH (10 Wt.%) sample, it has still higher compressive strength and Young’s modulus values compared to the pure PCL. The stress concentration and agglomeration of ceramic phases in the PCL matrix can be the main reason for the mentioned decline.

### *In Vitro* Degradation

**FIGURE 5:**
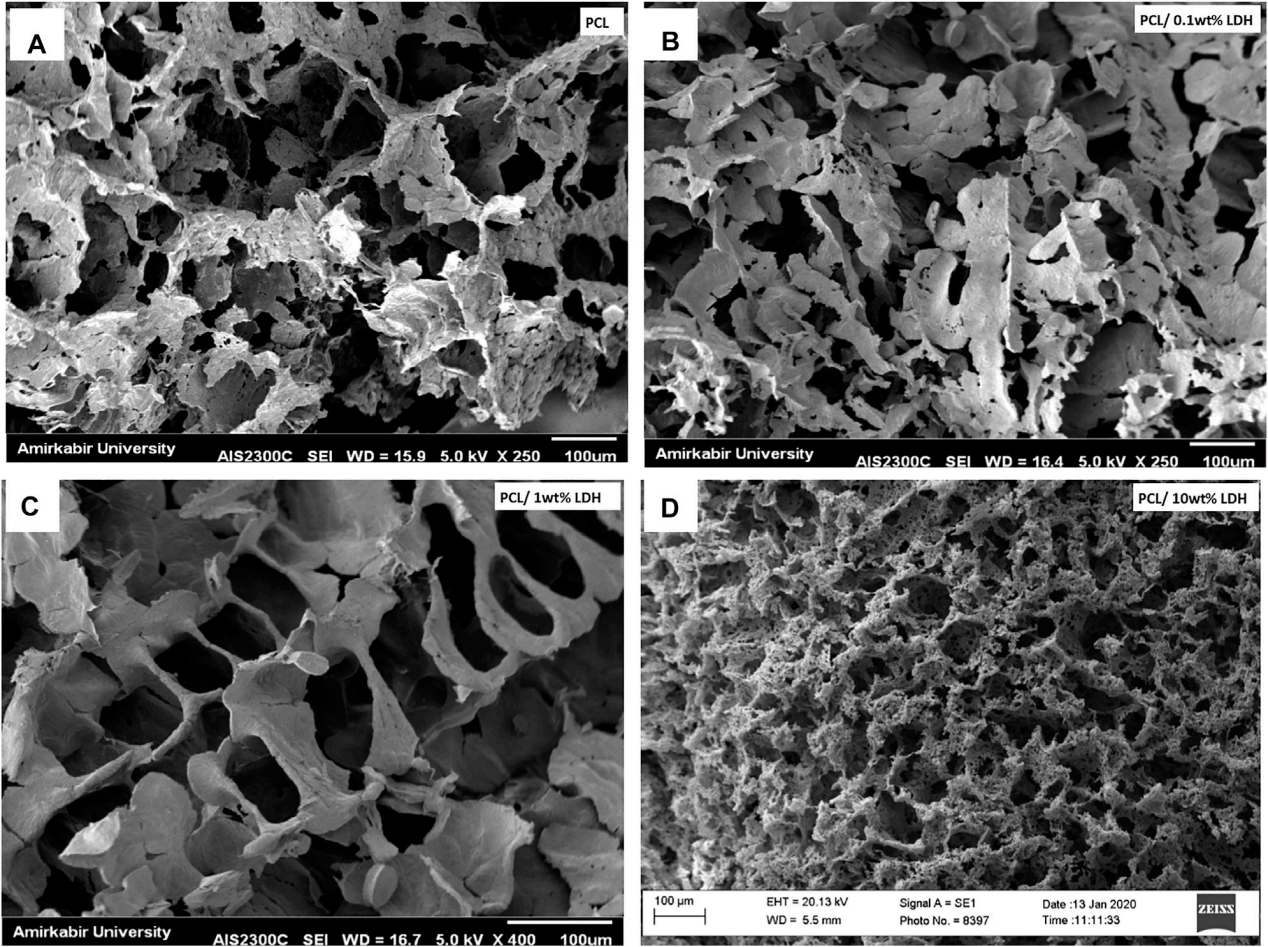
The effect of LDH addition on the degradation of porous nanocomposite PCL scaffolds and degrade via surface degradation. The bulk degradation of nanocomposite scaffolds was observed upon the addition of LDH. In comparison to the pure PCL samples, the scaffolds containing 10% nanoclay showed increased degradation, showing that nanoclay may accelerate both the adsorption of water inside the structure and the bulk breakdown of PCL. **(A)** PCL, **(B)** PCL/0.1 wt% LDH, **(C)** PCL/1 wt% LDH, **(D)** PCL/10 wt%LDH.

PCL predominantly degrades by ester cleavage of all available ester groups, which can occur from surface to bulk. Under the *in vivo* situation, PCL breakdown is a gradual procedure that takes more than 24 months to be completely degraded. Bone regeneration, on the other hand, is a reasonably quick process that takes more than 2 months to entirely replace tiny bone abnormalities (Pitt et al., 1981; Cipitria et al., 2011). As a consequence, an optimal scaffold for bone remodeling should have a quicker degradation rate and should adapt to the regrowth pace of injured tissue. As a side benefit, we would like a shorter degradation period for PCL-based scaffolds. The impact of LDH nanoclay content on the degrading characteristics of nanocomposite scaffolds was studied under accelerated settings using a 0.5M NaOH solution (Gaharwar et al., 2014). The inclusion of LDH nanoclay accelerates the degradation of the PCL. This might be attributed to PCL + LDH scaffolds’ reduced hydrophobicity as compared to PCL scaffolds. The addition of LDH may promote water adsorption, therefore exposing PCL chains to hydrolytic deterioration. After exposing the scaffolds to accelerated degradation for 72 h, microstructural features of the scaffolds were studied to evaluate the effect of LDH on the degradation process of the porous nanocomposite scaffolds (Figure 5). The addition of LDH nanoclay resulted in a different type of breakdown. For instance, after 72 h, a nanocomposite scaffold containing 0.1 Wt.% LDH showed a little fracture, but a sample containing 1 Wt.% exhibited considerable structural deterioration. Scaffolds completely deteriorated after 72 h in the sample containing 10Wt.% LDH, and the diameter and length of the pores had been significantly decreased. Adding LDH nanoclay to PCL increases the breakdown rate of the nanocomposite scaffolds and enables bulk degradation behavior. Overall, applying LDH to PCL increases the breakdown rate of the nanocomposite scaffolds and facilitates bulk degradation behavior.

### Thermal Analysis

**FIGURE 6:**
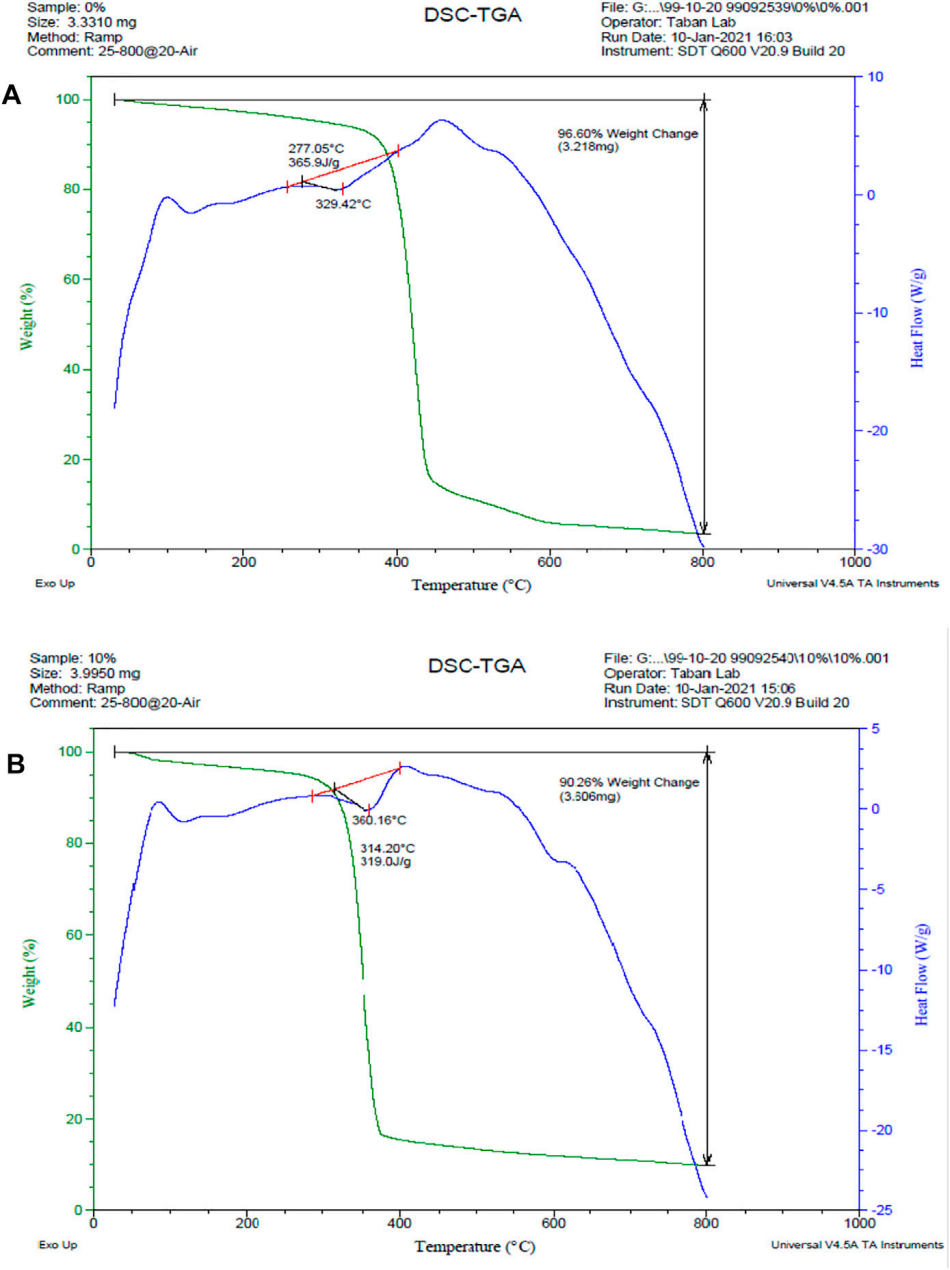
DSC/TGA diagrams. **(A)** PCL scaffold, **(B)** PCL + 10% LDH scaffolds. The decomposition temperature of polycaprolactone increased from 329 to 360°C with the addition of nanoclay, and the weight loss percentage has dropped from 96.6 % to 90.26%.

Polycaprolactone is a homopolymer with a melting point of 60°C. The decomposition temperature of polycaprolactone is 320–430°C, which has been changed by the addition of layered double hydroxide nanoclays (Persenaire et al., 2001). The results of the thermal analysis are shown in Figure 6. The decomposition temperature of polycaprolactone increased from 329 to 360° C with the addition of nanoclay, indicating an improvement in thermal stability, it can be seen in Figure 6B. The weight loss percentage has dropped from 96.6 % to 90.26%, as predicted, due to the presence of 10 wt.% nanoclay in the scaffold. Furthermore, the peak of the DSC diagram in the 600°C temperature range is due to the thermal degradation of LDH, which is not visible in the polycaprolactone graph.

### *In Vitro* Bioactivity

**FIGURE 7:**
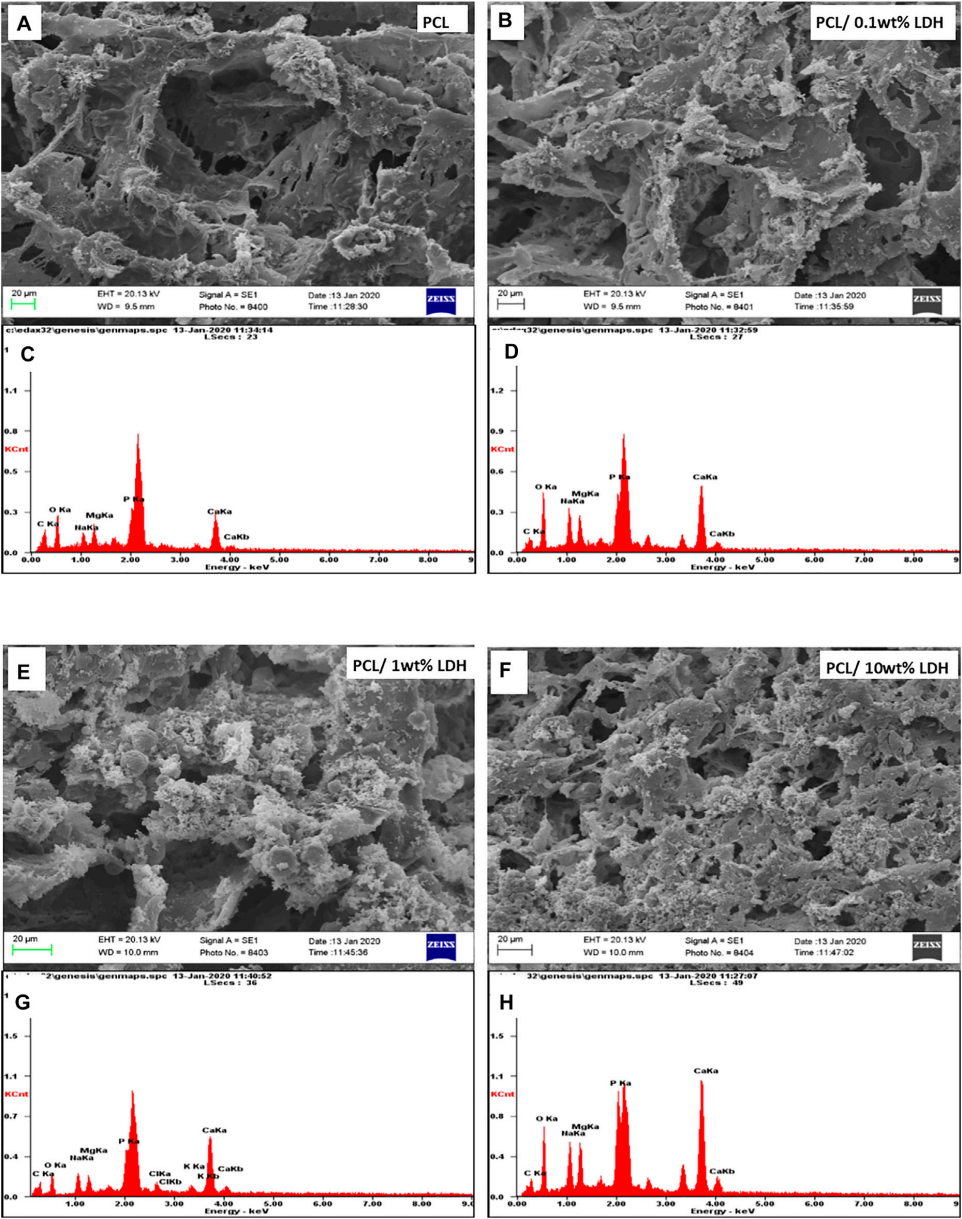
SEM micrographs of porous scaffolds after 7 days of soaking in SBF and The spectrum demonstrates peaks of carbon, oxygen, calcium, and phosphorus of each sample. **(A,C)** PCL, **(B,D)** PCL/0.1 wt% LDH, **(E,G)** PCL/1 wt% LDH, **(D,H)** PCL/10 wt% LDH.

The development of bone-bonding materials is a requirement for constructing artificial materials for bone tissue engineering. When exposed to SBF, the development of bone-like apatite on the surface of these materials is significantly associated with their *in vivo* bioactivity potential. Previous research has examined these features by exposing artificial biomaterials to SBF, an ionic liquid that closely approaches the composition of the body fluids. This technique can anticipate the synthesis of mineralized materials that could lead to the scaffold’s integration with the bone, which would be an indirect indication of osseointegration. On the other hand, when scaffolds were exposed to a highly saturated fluid, the increased roughness assists in the mineralization by offering nucleation sites for crystallization (Kokubo and Takadama, 2006). According to this point, SBF was used to examine the influence of LDHs on the *in vitro* biomineralization of nanocomposite porous scaffolds. The impact of LDH on the mineralization of porous PCL scaffolds was demonstrated in SEM images (Figure 7). The mineral deposition was found on pure PCL scaffolds, as predicted, and this finding is compatible with prior studies (Oyane et al., 2005). Cleavage of the ester linkages on the surface of PCL forms carboxylic (−COOH) and hydroxylic (-OH) functional groups during hydrolytic breakdown. The existence of these charged groups on the surface of PCL can act as a mimic for the proteins found in bone tissue, stimulating mineralization. The negatively charged groups on the surface of PCL attract calcium ions to the surface of scaffolds, facilitating the production of hydroxyapatite crystals (LeGeros, 2008; Jaiswal et al., 2012). EDX analysis was used to assess the elemental composition of the sample surfaces following immersion in SBF. As it can be seen in Figure 7, all the samples revealed mineralized apatite layers formed on their surfaces. According to the EDAX diagram, adding LDHs to porous scaffolds considerably increases the number of mineralized deposits. It was observed that both PCL and PCL + LDH scaffolds show strong phosphate and calcium peaks. Mineral deposits covering the porous scaffold were found at higher LDH concentrations (10 Wt.% LDH). Fortunately, PCL + LDH nanocomposites have been discovered to have high apatite deposition potential. According to previous studies, apatite has a high affinity for serum proteins and growth hormones, which might help hBMSCs proliferate, differentiate and express genes (Wu et al., 2015).

### Mineralization Study

**FIGURE 8:**
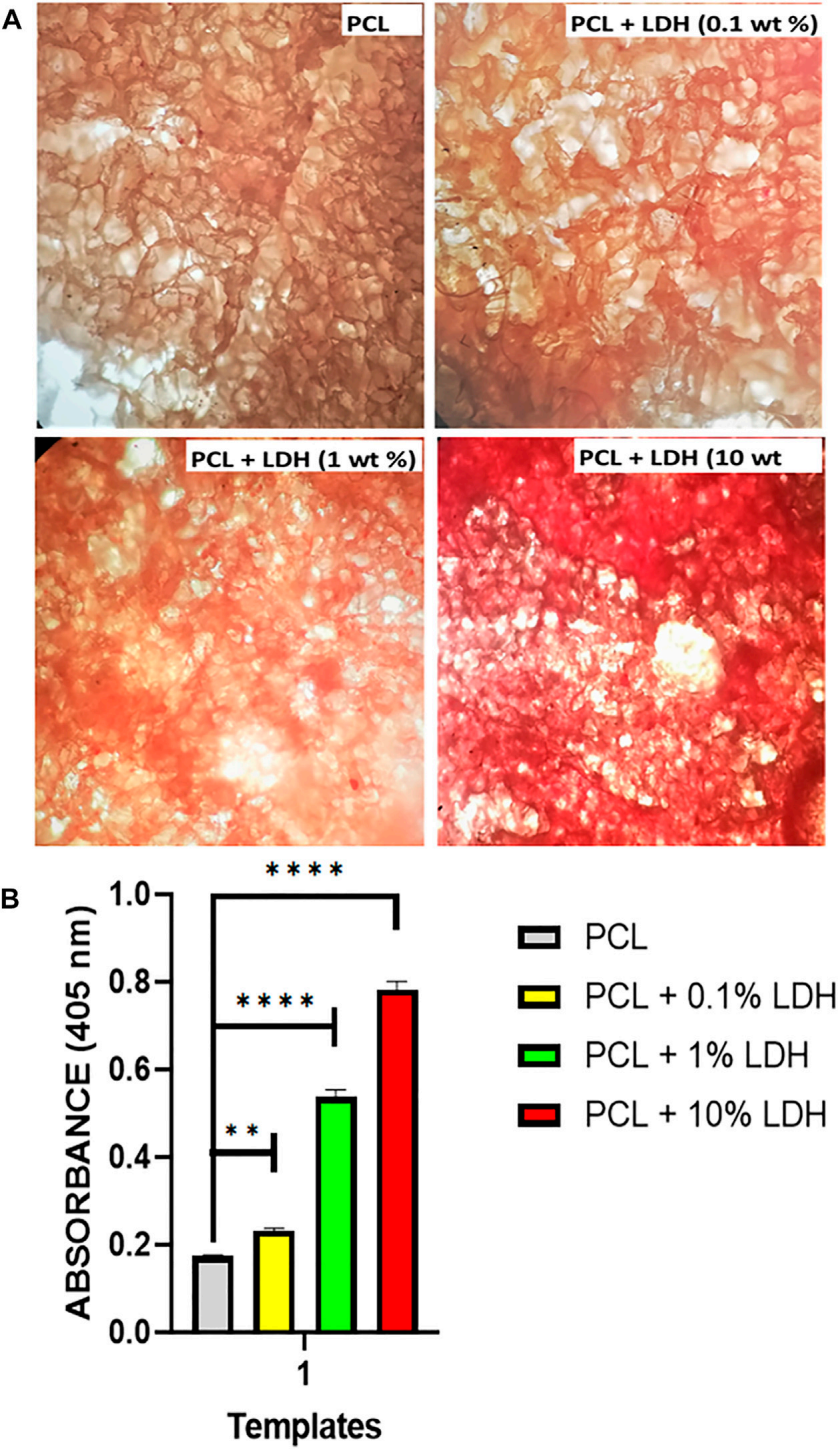
Nanoclay effects on the development of mineralized matrices. **(A)** On day 14, alizarin red staining was used to assess the mineralized matrix’s production. The results showed that in scaffolds loaded with LDH, a higher amount of mineralized matrix was observed compared to PCL scaffolds. This reveals that the nanoclay in the polymeric scaffold initiates and maintains stem cell osteogenic differentiation. **(B)** According to the image quantification, the presence of nanoclay resulted in increased mineralized matrix coverage. Significant levels are **p ≤ 0.01 and ****p ≤ 0.0001.

To measure the amount of mineralization on each of the formulations under consideration, we studied the deposition of inorganic calcium, which is a sign of thorough stability and maturation of differentiated cells. The mineralized matrix formation on the PCL scaffolds could be assessed by Alizarin Red, which specifically stains for calcium ions (Figure 8A). The red staining was detected in all of the formulations, but the LDH scaffolds had an intense dark red coloring, indicating that hBMSCs experience increased osteogenic differentiation when LDH is added to the PCL. Because surface-mediated ion-exchange allows LDH composites to release calcium ions, it has a significant influence on the quantity of calcium accumulation. By evaluating the extent of the stained zone, we were able to quantify the amount of mineralized matrix (Figure 8B). As compared to all other scaffolds, the scaffold containing 10 Wt.% LDH had the largest area fraction of stained region, whereas scaffolds using 0.1 Wt.% LDH had mineralized areas roughly similar to the pure PCL. The PCL biocomposite has been proven to promote osteogenic differentiation in previous investigations (ShiraliPour et al., 2021). They discovered that the development of mineralized matrix is aided by the nanocomposite PCL scaffold. The inclusion of bioactive nanoparticles into the biopolymer PCL was found to promote and reinforce osteogenic differentiation of hBMSCs. When compared to PCL scaffolds separately, our findings show that adding LDH nanoclay to PCL maintained and improved the osteogenic differentiation of seeded hBMSCs.

### Cell Viability and Metabolic Activity

**FIGURE 9:**
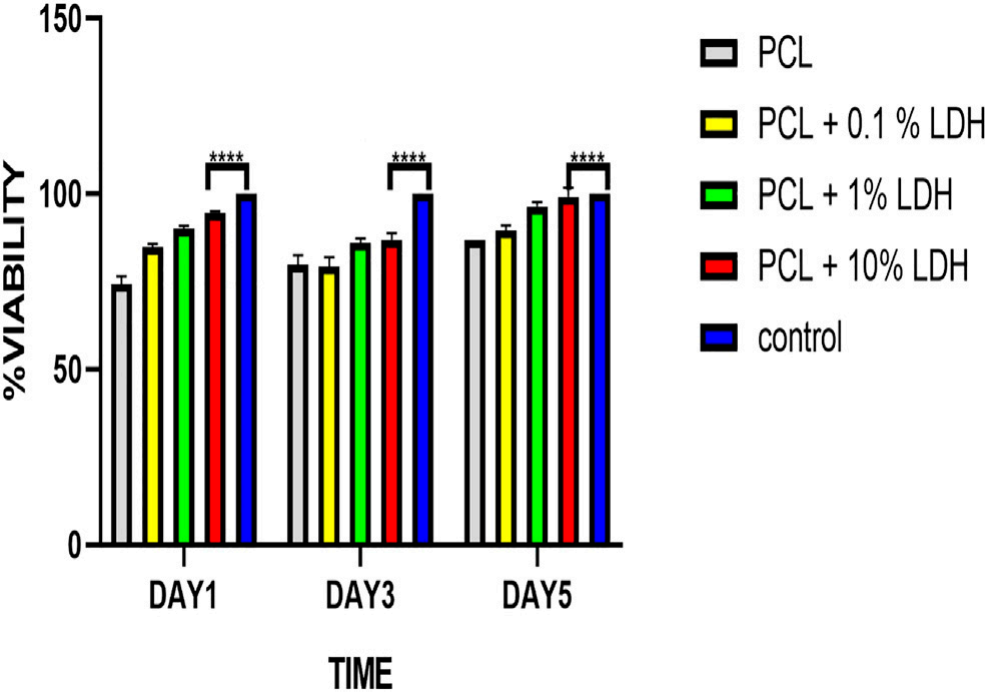
Cell viability results of PCL, PCL/LDH (0.1 wt%), PCL/LDH (1 wt%), and PCL-LDH (10%) porous scaffolds after 1, 3, and 5 days of culture by MTT assay. The control is hBMSCs cultured on tissue cultured polystyrene (TCPS). ****p ≤ 0.0001.

The MTT assay was used to investigate the influence of scaffolds on cellular viability and metabolic activity (Hosseini et al., 2019). After 1, 3, and 5 days of culture, the cell viability result of the MTT experiment was indicated. From day one to day 5, all four groups’ cell numbers grew, and all samples showed elevated cellular proliferation. The vitality and proliferation of hBMSCs seeded on enriched LDH scaffolds were significantly improved as compared to the pure PCL scaffold and control (Figure 9). In this regard, the cells attached to the surface of scaffolds with various LDH levels ranging from 0.1 wt.% to 10 wt.% grew rapidly, demonstrating a cell-friendly microenvironment. The positively charged LDH porous nanocomposite allows cells to adhere and grow by allowing them to interact with the negatively charged cell membranes. In comparison to other samples, the PCL + LDH (10 Wt.%) sample showed the greatest vitality and proliferation (*p* < 0.05). The surface chemistry of LDH may be responsible for this beneficial effect due to positively charged LDH nanoparticles help cells to adhere and grow by allowing them to associate with negatively charged cell membranes, and besides, the results showed that all scaffolds provided acceptable cellular microenvironments in which the cell’s activities could be preserved. Even in the PCL + LDH (10 Wt.%) sample, no cytotoxic effect attributable to the presence of LDH was observed.

### Cell Attachment Study

**FIGURE 10:**
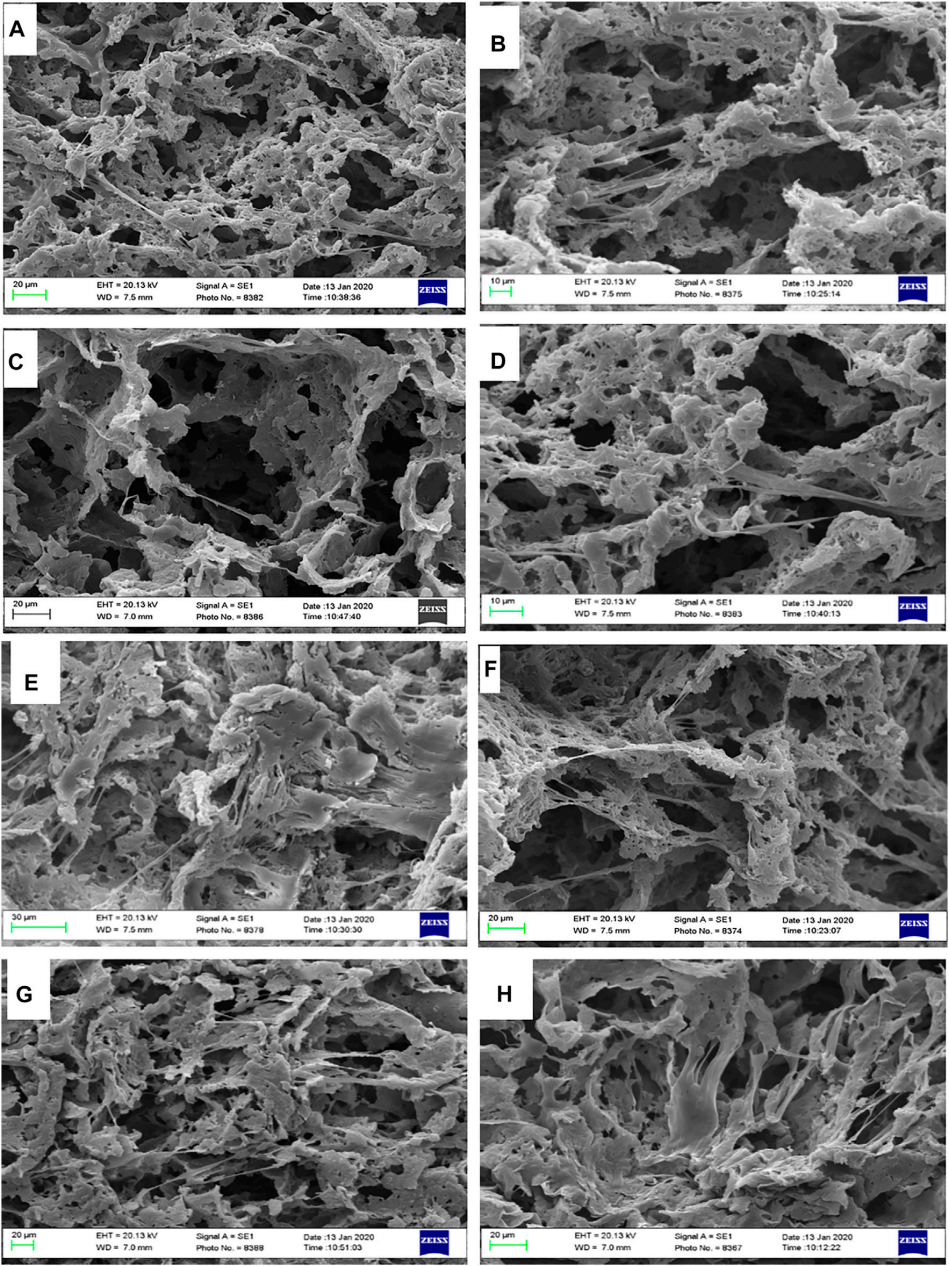
SEM observation of hBMSCs cultured on highly porous biocomposite scaffolds. **(A,B)** PCL, **(C,D)** PCL + 0.1% LDH, **(E,F)** PCL + 1% LDH, and **(G,H)** PCL + 10% LDH scaffolds.

SEM micrographs revealed excellent cell adherence and cell growth within the porous scaffolds. Cells have adhered effectively to the structure’s wall and have entered and covered the pores, as can be seen in SEM pictures (Figure 10). The ECM was also observed to be aligned with the pores. Porous nanocomposite scaffolds’ resemblance in structure and composition to the natural bone can be a potent inducer of cell growth. Furthermore, the cell culture study results showed that LDH nanoclay is a safe material to employ since it enhanced cell adhesion and proliferation (Baradaran et al., 2020). Additionally, the presence of LDH nanoparticles in the scaffold can lead to the proper cell interaction, resulting in a perfect platform for the formation of cell colonies. Previous research has also shown that adding nanoparticles to the matrix can improve cellular connections and behavior (Gaharwar et al., 2014). Generally, there could be found some factors that might influence initial cell attachment, including protein absorption capability, surface free energy, roughness, and chemical characteristics of the surface (ShiraliPour et al., 2021). It is clearly shown in the Figure 10, all nanocomposite scaffolds could adequately promote cellular adhesion. Mesenchymal stem cells were evenly distributed throughout all of the porous scaffolds, and the spindle-like morphologies of the cells revealed that a satisfactory environment for cell colonization was present. The hBMSCs have formed numerous networks within the scaffolding’s various pores, which substantially facilitates their development and expansion. The flattened cells with random patterns were also found to be surrounded by an extracellular matrix.

### Gene Expression

**FIGURE 11:**
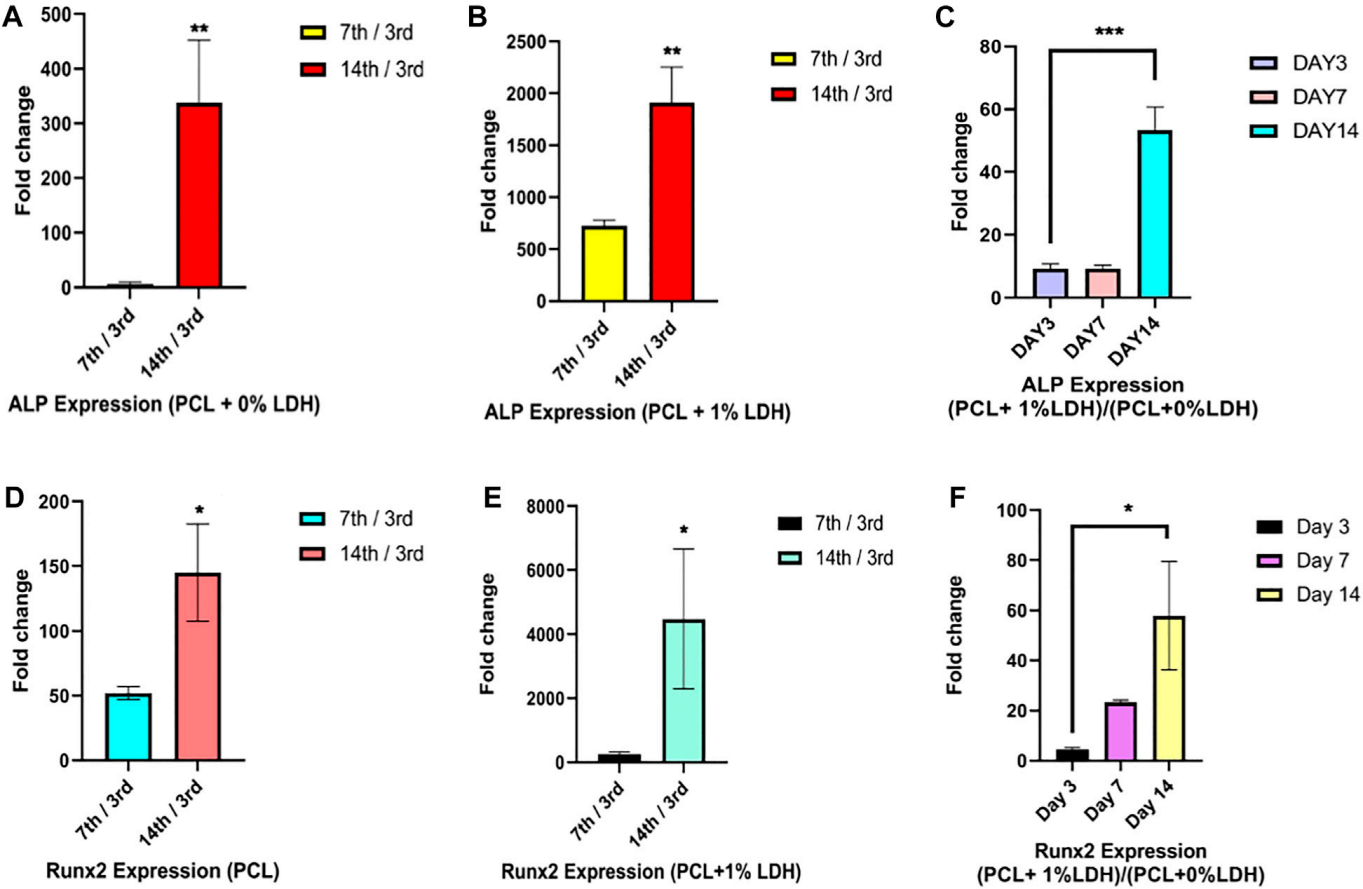
Real-Time RT‐PCR assay of ALP and RNX2 genes expression in hBMSCs. **(A,D)** evaluation of ALP and RUNX2 gene expression in hBMSCs seeded on PCL on days 7 and 14 compared to day 3. **(B,E)** evaluation of ALP and RUNX2 gene expression in hBMSCs seeded on PCL + LDH (1 wt%) on days 7 and 14 compared to day 3, and **(C,F)** evaluation of ALP and RUNX2 gene expression in hBMSCs cultured on PCL/LDH (1 wt%) compared to pure PCL at Days 3, 7, and 14. Significant levels are *p ≤ 0.05; **p ≤ 0.01 and ***p ≤ 0.001.

The osteogenic differentiation of the hBMSCs cultured on PCL and PCL + LDH (1Wt.%) was performed using real-time RT-PCR at days 3, 7, and 14 (Figure 11). Two significant osteogenic-related gene markers, including Runx2, and ALP were considered for assessment in samples as primary and maturation stage markers of the differentiation process, respectively. The level of Runx2 expression in hBMSCs cultured on PCL and PCL + LDH (1 Wt.%) groups was not different between Day 3. However, this difference was increased on day 7 and more significantly on Day 14. Also, Runx2 was expressed in the PCL + LDH (1Wt.%) group more significant than the pure PCL group. The ALP expression pattern was not different between PCL + LDH (1Wt.%) groups on days 3 and 7, but this difference was increased on day 14 significantly, whereas, in the PCL + LDH (1 Wt.%) samples, it was remarkably higher than the pure PCL group.

## Discussion

Orthopedic diseases, injuries, and bone tissue fractures are becoming more common health issue. The demand for tissue replacement and therapy has grown due to the growing prevalence of these illnesses (Leong, 2004). The design of scaffolds that best mimic bone characteristics are an essential aspect of bone tissue engineering. Three-dimensional porous scaffolds offer the first environment for mesenchymal stem cells to adhere, proliferate, and differentiate. Bone tissue engineering has been found to induce calcification and angiogenesis, modify the immune response to injuries and fractures, enhance implant biocompatibility, attack or prevent infection, and fill big gaps which were produced by the lack of traumatic bone (Hosseini et al., 2019). There could be found certain aspects of scaffold design such as architecture, porosity, morphology, biochemistry, mechanical characteristics, and material degradation rates which should be taken into account. Furthermore, an ideal scaffold should promote a milieu in which cells may migrate and proliferate, as well as generate the new matrix necessary for the regrowth of new bone (Mozaffari et al., 2019). As previously indicated, the purpose of this study is to create a highly porous nanocomposite scaffold based on PCL + LDH using the TIPS method. One of the most significant approaches for fabricating scaffolds in bone tissue regeneration is the TIPS method which provides a highly porous structure with varied pores that leads to migration, proliferation, and cells adhesion (Gandolfi et al., 2019). The percentage of porosity, toxicity, mechanical and thermal properties of nanocomposite scaffolds were assessed as LDH was increased. Additionally, osteoconductivity of the scaffolds was examined in regards to hBMSCs differentiation. LDH was effectively synthesized in the first stage using the co-precipitation technique. Following that, thermally induced phase separation was used to manufacture PCL composite scaffolds with varied LDH contents, as validated by XRD analysis. Polymers on their own cannot provide all of the properties of an ideal scaffold. Their utilization as composite scaffolds in combination with bioactive ceramics makes them an acceptable and feasible scaffolding option. In this experiment, bioactive ceramic (LDH) was chosen as a polymer scaffold enhancer due to the low degradability rate, poor mechanical characteristics, and hydrophobicity of polycaprolactone. The LDHs utilized in the polymer scaffold are incorporated as a ceramic phase in the scaffold’s construction. This nanoclay increases the hydrophilicity and roughness of the scaffold surface, resulting in more and better cell connections and stimulating and activating the bone differentiation cascade (Makwana et al., 2020). According to earlier studies, the construction of scaffolds based on polycaprolactone shows that their purpose was to increase the porosity and enhance scaffolds’ mechanical properties to strengthen their structural and biological properties (Shafiei et al., 2016; Gandolfi et al., 2019; Baradaran et al., 2020). In this current research, human mesenchymal stem cells were seeded on PCL + LDH nanocomposite, which was produced using the TIPS technique. As essential features of bone differentiation, the accuracy of differentiation of these cells into bone was tested using a qualitative and quantitative alizarin red test and the expression of RUNX2 and ALP bone genes using real-time PCR. All these activities have not been studied in previous papers so far. Porosity is an important feature of BTE that influences the biological characteristics of scaffolds. Mechanical properties are reasonable when the porosity degree is suitable, and the pore diameters are acceptable. Porosity permits cells to connect and migrate while also allowing nutrients and biomolecules to pass through. Osteoblasts are around 10–50 µm in size. However, bigger holes (100–200 µm) are preferred by osteoblasts for renewing mineralized bone following implantation (Sugawara et al., 2005). These pores allow cells to infiltrate and participate in colonization, migration, and vascularization *in vivo*. Pore sizes of less than 100 µm are linked to the development of non-mineralized osteoid or fibrous tissue (Iviglia et al., 2019). Previous research has revealed that small hole size limits cell penetration *in vitro*, even though pores larger than 50 µm (macropores) have favorable impacts on osteogenic quality. Pores with a diameter of less than 10 µm (micropores) have a larger surface area, which promotes bone protein adsorption and ion exchange (Diaz-Rodriguez et al., 2018; Morejón et al., 2019). The SEM micrographs of scaffolds confirmed that all samples exhibited a highly porous structure with various pore sizes which show a wide correlation between porosities and by increasing LDH concentration, no changes in porosity were observed. These pores have been estimated by ImageJ to be less than 1 μm in diameter and even more than 100 μm in diameter which proved to be a suitable bone tissue engineering scaffold. One of the main functions of bone tissue in the body is to bear weight and pressure, so high-pressure tolerance is one of the main characteristics of bone scaffolds. As a result, significant attention must be taken, and mechanical characteristics must be thoroughly studied for developing scaffolds for optimal load and pressure transmission to the target tissue (Halling Linder et al., 2016). Polycaprolactone usage within tissue engineering is restricted to non-load-bearing defects, even though it has a slew of advantages. The weak mechanical characteristic of PCL continues to be a serious issue for BTE researchers. The mechanical characteristics of the scaffolds are predicted to improve when the PCL polymer phase and the LDH ceramic phase are combined. Because the LDH load is positive and the PCL load is negative, a uniform distribution of LDH and PCL bonding enhances the scaffold’s compressive strength. As the level of LDH increases, young’s modulus rises. The PCL + 1 Wt.% LDH scaffold has the highest young’s modulus (1.3251 MPa) and compressive strength among all the produced scaffolds. This amount of LDH is the optimum for improving the scaffold’s strength and compressive strength. While in sample 10Wt.% LDH, as the density of nanoclay in the scaffold increases, the scaffold’s flexibility decreases, resulting in the scaffold’s fragility under pressure. A previous study has shown that young’s modulus of the spinal bone is considered 1.1–3.9 MPa. As a consequence, PCL + LDH (1 Wt.%) might be the most appropriate sample for bone tissue engineering applications (Lakatos et al., 2014). The results of thermal analysis have indicated that the addition of nanoclay phase increases the thermal stability of the polymer because it may affect the crystallinity as well as the electrostatic forces of the polymer. The weight loss diagrams have shown that the residual weight in the sample containing nanoclay is higher because there could be found minerals in the nanoclay structure, whereas the polymer degrades completely at lower temperatures, and only carbon remains. One of the reasons for the increase in thermal stability by LDH mentioned in the sources is its layered structure, which prevents the release of evaporative materials and increases the decomposition temperature (Krishna and Pugazhenthi, 2013; Bagheri and Mahmoodzadeh, 2020). The goal of tissue engineering is to create scaffolds that perfectly match the characteristics of the body’s main and natural tissues. The formation of hydroxyapatite layers, for instance, is used in bone tissue engineering to approach bone structure. For this purpose, scaffolds were placed in the body simulator solution, SBF, to facilitate the ossification process. Inorganic mineral particles have been recognized in all samples, even pure PCL scaffolds. As reported in previous studies, mineral sediments on pure PCL scaffolding are due to hydrolytic degradation of PCL, which is mainly caused by severing ester bonds on the surface of PCL fibers, which leads to the formation of a negative charge, carboxyl groups (-COOH) and hydroxyl (-OH). The basis of mineral sediments and their growth is the adsorption of calcium cations by negatively charged groups on the surface of scaffolds, which leads to the creation of hydroxyapatite crystals in the form of PO_4_ (Pitt et al., 1981; Kokubo, 1991; Persenaire et al., 2001; Bravo-Suárez et al., 2004; Leong, 2004; Oyane et al., 2005; Sugawara et al., 2005; Kokubo and Takadama, 2006; LeGeros, 2008; Guarino and Ambrosio, 2010; Yang et al., 2010; Cipitria et al., 2011; Fayyazbakhsh et al., 2012; Jaiswal et al., 2012; Krishna and Pugazhenthi, 2013; Gaharwar et al., 2014; Lakatos et al., 2014; Li et al., 2014; Wu et al., 2015; Halling Linder et al., 2016; Shafiei et al., 2016; Conoscenti et al., 2017; Kino et al., 2017; Mohammadi et al., 2017; Diaz-Rodriguez et al., 2018; Gandolfi et al., 2019; Hosseini et al., 2019; Iviglia et al., 2019; Morejón et al., 2019; Mozaffari et al., 2019; Tohidlou et al., 2019; Bagheri and Mahmoodzadeh, 2020; Baradaran et al., 2020; Makwana et al., 2020; ShiraliPour et al., 2021). Additionally, Many previous investigations have shown that LDH plays a significant role in osteogenesis and mineralization (Baradaran et al., 2020; Cheng et al., 2021). It was also observed in this study that the rate of mineralization of scaffolds increased as the ceramic phase of LDH in their structure increased. Overall, LDH nonclay and osteogenesis show a positive correlation. Cell adhesion is the procedure by which cells attach to a platform, often known as the extracellular matrix. Roughness, hydrophilicity, chemistry, and the surface charge, all, have a role in this process. In addition, as previously noted concerning LDH’s involvement in increasing cell adherence to the surface, the roughness and porosity of the surface are also essential. LDH, a bioactive substance, has a significant impact on cellular activity and stem cell development. The adhering cells spread properly on the surface of the nanocomposite and bridged between pores, as seen in Figure 10. Cells were also detected penetrating the holes. It is well realized that pore size has a substantial impact on cell activity. Therefore, porous structures are better for tissue engineering. Hydrotalcite-like, double-layered hydroxide ceramics are known as osteogenic stimulus structures. To increase the osteoinductive behavior of the host tissue, it can balance between different chemical and physical properties (Fayyazbakhsh et al., 2012). *In vitro* studies have indicated that LDH has had an effective role in cellular signaling, particularly in activating the MAPK and Rac1 signaling pathways. LDH stimulates pre-osteoblast cells by first activating the MAPK pathway, the JNK, and ERK, and ultimately phosphorylating CREB in the nucleus, resulting in the activation of the bone genes RUNX2, ALP, and OSX. By expressing bone genes, OPG and RANKL proteins are expressed. These proteins are found in equal amounts in mononuclear cells and promote the formation of specialized multinucleated osteoclasts, which are responsible for bone resorption. OPG and RANKL create a dynamic scaffold. In general, the data show that LDH is involved in bone regeneration by interfering with osteoblasts and osteoclasts (Kang et al., 2018). The expression of bone genes (ALP, RUNX2) at the RNA level was assessed using Real-time molecular PCR with the goal of further extensive research to verify the distinguishing ability of scaffolds. The bioactivity and considerable effect of LDH in differentiating stem cells towards the bone line are confirmed by a significant increase in the expression of ALP and RUNX2 genes in the optimum sample (1 Wt.%LDH) compared to the control sample.

## Conclusion

In this investigation, highly porous PCL + LDH nanocomposite scaffolds were fabricated using the TIPS method. By changing the LDH concentration, a series of nanocomposite samples were created. According to SEM micrographs, the micropores were in the size range from 5 to 150 μm, which were appropriate for cell adhesion and proliferation. When PCL scaffolds were enriched with LDH nanoclay and exposed to the SBF, it stimulated *in vitro* biomineralization, showing that hybrid scaffolds have bioactive properties. The results indicated that highly porous nanocomposite scaffolds (greater than 90%) were produced by the TIPS method. Adding LDH to PCL scaffolds had no significant effect on the porosity percentage. The results of MTT tests revealed that PCL + LDH was biocompatible and non-toxic. The use of LDH nanoclay enhanced the degradation rate of PCL. The inclusion of LDH nanoclay into the porous scaffolds greatly improves hBMSCs adhesion, proliferation. According to the results, PCL + LDH nanocomposite scaffolds with osteoconductivity characteristics have a great potential for osteogenic differentiation, and the osteogenic differentiation ability of human MSCs was enhanced when cultured on PCL+1% LDH compared to the pure PCL (over 50-fold increase). Overall, highly porous nanocomposite PCL + LDH might be a potential candidate for treating bone fractures, injuries, and defects in bone tissue engineering applications.

## Data Availability

The raw data supporting the conclusion of this article will be made available by the authors, without undue reservation.
